# Inoculation with a microbial consortium increases soil microbial diversity and improves agronomic traits of tomato under water and nitrogen deficiency

**DOI:** 10.3389/fpls.2023.1304627

**Published:** 2023-12-06

**Authors:** Valerio Cirillo, Ida Romano, Sheridan L. Woo, Emilio Di Stasio, Nadia Lombardi, Ernesto Comite, Olimpia Pepe, Valeria Ventorino, Albino Maggio

**Affiliations:** ^1^Department of Agricultural Sciences, University of Naples Federico II, Portici, Italy; ^2^Department of Pharmacy, University of Naples Federico II, Naples, Italy; ^3^National Research Council, Institute for Sustainable Plant Protection, Portici, Italy; ^4^Task Force on Microbiome Studies, University of Naples Federico II, Portici, Italy

**Keywords:** *Azotobacter chroococcum*, *Trichoderma afroharzianum*, nutrient stress, water stress, biostimulants, tomato rhizosphere

## Abstract

Microbial-based biostimulants, functioning as biotic and abiotic stress protectants and growth enhancers, are becoming increasingly important in agriculture also in the context of climate change. The search for new products that can help reduce chemical inputs under a variety of field conditions is the new challenge. In this study, we tested whether the combination of two microbial growth enhancers with complementary modes of action, *Azotobacter chroococcum* 76A and *Trichoderma afroharzianum* T22, could facilitate tomato adaptation to a 30% reduction of optimal water and nitrogen requirements. The microbial inoculum increased tomato yield (+48.5%) under optimal water and nutrient conditions. In addition, the microbial application improved leaf water potential under stress conditions (+9.5%), decreased the overall leaf temperature (-4.6%), and increased shoot fresh weight (+15%), indicating that this consortium could act as a positive regulator of plant water relations under limited water and nitrogen availability. A significant increase in microbial populations in the rhizosphere with applications of *A. chroococcum* 76A and *T. afroharzianum* T22 under stress conditions, suggested that these inoculants could enhance soil microbial abundance, including the abundance of native beneficial microorganisms. Sampling time, limited water and nitrogen regimes and microbial inoculations all affected bacterial and fungal populations in the rhizospheric soil. Overall, these results indicated that the selected microbial consortium could function as plant growth enhancer and stress protectant, possibly by triggering adaptation mechanisms via functional changes in the soil microbial diversity and relative abundance.

## 1 Introduction

The global food demand is anticipated to increase from 35% to 56% in the period of 2010 to 2050, while the population at risk of hunger is projected to increase to +8% over the same time frame (van Dijk et al., 2021). To maintain a high quantity of crop production and reduce yield loss, chemical products (fertilizers, pesticides, herbicides, etc.), hormones and antibiotics are commonly used in agriculture (Savci, 2012; Pathak, 2018; Gangwar et al., 2023). Concerns over human and environmental health and negative impacts arising from chemical residues in soil, water, and food as well as exposure risks by farm workers have received considerable attention. As a consequence, in the last two decades, the scientific community is looking for innovative and eco-sustainable strategies to increase agricultural production, meet food needs, and reduce environmental impact (Comite et al., 2021; Silletti et al., 2021). The use of microbial inoculants as agricultural-probiotics, is an attractive environmental-friendly alternative strategy to agro-chemical inputs to ensure crop yield and quality (Fiorentino et al., 2018; Woo and Pepe, 2018). Probiotics are living microorganisms that offer benefits to the host plant by providing nutritional inputs, protection from pathogen-pest attack, improved fitness, enhanced growth and health also in stress conditions (Hossain et al., 2017; Van Oosten et al., 2017; Romano et al., 2020b). Biostimulant formulations containing beneficial microorganisms and/or natural substances (e.g., humic acids, seaweed and plant extracts, protein hydrolysate and silicon) can stimulate plant vigor, growth, and yield, even under sub-optimal growth conditions (Viscardi et al., 2016; Sheridan et al., 2017; Di Stasio et al., 2020; Comite et al., 2021).

Among beneficial microorganisms, plant growth promoting rhizobacteria (PGPR) emerge as key players in agricultural microbial applications, noted for their positive effects on plant growth, by favoring the absorption of nutrients, such as nitrogen and phosphate (Ventorino et al., 2007; Reddy, 2013; Ahemad and Kibret, 2014). These bacteria are commonly represented by genera such as *Bacillus*, *Pseudomonas*, *Azospirillum*, *Azotobacter*, *Alcaligens*, *Arthobacter*, *Agrobacterium* and *Rhizobium*. The nitrogen-fixer *Azotobacter* is a free-living aerobic rhizobacterium, that can stimulate plant growth through nutrient supplementation or through the production of phytohormones such as auxins, gibberellins, and cytokinins (Viscardi et al., 2016; Wani et al., 2016; Van Oosten et al., 2018), as well as the production of large quantities of exopolysaccharides (Ventorino et al., 2019a; Romano et al., 2020b). Members belonging to this genus are involved in nutrient processes such as nitrogen cycling, phosphate solubilization (Wani et al., 2013), mobilization of iron (Rizvi and Khan, 2018) and the biodegradation of many commonly used chemical pesticides (Gurikar et al., 2016). *Azotobacter chroococcum* is a promising candidate for improvement of plant growth and abiotic stress tolerance (Viscardi et al., 2016; Silletti et al., 2021). Extending the biodiversity of beneficial microorganisms, it has been proven that selected fungal strains of *Trichoderma* also have PGPR-like effects, and establish positive interactions with plants including biological control, plant growth promotion, and induced plant resistance (Harman et al., 2004; Shoresh and Harman, 2008; Raaijmakers et al., 2009; Woo et al., 2023). *Trichoderma* spp. and endophytic fungi have become prominent on the agricultural scene, due to their multiple positive effects and improvement in yield properties of given crops (Harman et al., 2004; Lorito and Woo, 2015; Woo et al., 2023). *Trichoderma* spp. produce over 250 metabolic products, including secondary metabolites, peptides, proteins, and cell-wall-degrading enzymes with biostimulant or protective effects on plants (Woo and Pepe, 2018; Vinale and Sivasithamparam, 2020). Furthermore, plants inoculated with *Trichoderma* have also demonstrated effective mitigation of the negative consequences of drought stress by improving proline concentration in plant tissue and the synthesis of growth hormones (Mona et al., 2017). Considering that diverse microbial based-biostimulants are able to provide stress protection via diverse mechanisms of action (Yakhin et al., 2017), the combination of two or more selected strains can be proposed to enhance their action (Rouphael and Colla, 2018; Gemin et al., 2019).

Previous work has reported the use of microbial consortia containing both rhizobacteria and fungi as a sustainable technique for the maintenance of soil health and the increase of crop productivity (Carneiro et al., 2023). One of the main benefits of their integrated use includes the reduction in the need for water and fertilizer applications, which provides a dual benefit: i) reduced economic production costs through more efficient and resilient farming systems; ii) decreased environmental impact, due to lower contamination with biological products when compared to mineral fertilizers (Carneiro et al., 2023). Innovative microbial consortia can include *Trichoderma* strains in combinations with plant-beneficial microorganisms such as *Azotobacter* (Woo and Pepe, 2018; Woo et al., 2023). Several studies highlighted the versatile and beneficial effects of combined *Azotobacter* and *Trichoderma* inoculation on improving crop performance across diverse environmental and nutrient conditions. This synergistic interaction extends to the formation of *Trichoderma*-*Azotobacter* biofilm, positively impacting soil nutrient availability and overall plant growth in wheat, cotton, and chickpea (Velmourougane et al., 2017; Velmourougane et al., 2019). Despite their extensive use in agriculture, microbial-based biostimulants have mainly been tested with a focus on improving crop yield and quality aspects (du Jardin, 2015), whereas their potential role on crops exposed to biotic and/or abiotic stress needs to be further investigated. The contribution of microbial-based biostimulants as abiotic stress protectants and growth enhancers is becoming increasingly more important, also in the context of climate change, which is exacerbating the outbreak of pests and diseases (Rosenzweig et al., 2001), as well as crop exposure to extreme temperatures, drought, and soil salinization (Ahuja et al., 2010; Cirillo et al., 2018) which all have a strong negative impact on crop yield and quality.

In this work, we assessed the function of the microbial consortium containing the bacterium *Azotobacter chroococcum* 76A and the fungus *Trichoderma afroharzianum* T22 on tomato (*Solanum lycopersicum* L.) crop subjected to water and nitrogen deficiency. We hypothesized that co-inoculation of the tomato root system with these two microorganisms could facilitate plant tolerance to a combination of water and nutrient stress due to their known complementary modes of action on the host crop. Furthermore, the effects of this microbial consortium on tomato yield and fruit qualitative, as well as the influence on the surrounding soil microbial community were also assessed. These findings could help to understand the functional link between the main components of this microbial-based biostimulant and the modulation of tomato plant response to the combined abiotic stressors.

## 2 Materials and methods

### 2.1 Experimental design and sampling

A field experiment with tomato (*Solanum lycopersicum* L.) was conducted at the experimental farm of the University of Naples Federico II, located at Bellizzi, Italy (lat. 43°31’N, long. 14°58’ E; alt. 60 m above sea level) on sub-alkaline soil (pH 7.5), silty-clay-loam (Clay 334 g kg ^-1^, Silt 241 g kg ^-1^, Sand 425 g kg ^-1^) with low nitrogen and soil organic matter (1.2 g kg^-1^ and 18.4 g kg^-1^, respectively). The meteorological data during the experiment are reported in **Figure S1**. Field plots for all treatments were moldboard plowed at 30 cm depth, followed by secondary tillage with a soil grubber and harrow for seedbed and transplanting preparation.

Tomato seeds cv. Vulcan F1 (Nunhems^®^—Bayer, Leverkusen, Germany) were germinated in peat planting trays and grown in the greenhouse until the 3^rd^–4^th^ true leaf. Plants were transplanted with a plant density of 3.3 plants per m^2^ and irrigated, starting with drip lines with emitters of 1.5 L h^-1^ flow, 0.3 m apart. The experiment was arranged in a randomized block design in plots of 50 m^2^ with three replicates. Plants were treated with a microbial consortium (T) as below described, and non inoculated plants were used as controls. Nutritional input (I) included two levels of nitrogen (N) fertilization (optimal: 100%; and sub-optimal: 70% of estimated plant N requirements). Nutrients were supplied via fertigation during the whole crop cycle, providing the plant with 104 N, 124 P_2_O_5_ and 122 K_2_O units ha^-1^ for the optimal N treatment, and 73 N, 124 P_2_O_5_ and 122 K_2_O units ha^-1^ in the sub-optimal N plots. The fertilizers used were ammonium nitrate and potassium monophosphate. In order to impose water stress, plants were irrigated with 70% (sub-optimal, moderate stress) of the optimal water supply (100%), as estimated with the FAO-24 Pan method.

### 2.2 Microbial strains, inoculum preparation and tomato treatments

The microbial biostimulant treatment (A+T) used two different microorganisms: *Azotobacter chroococcum* strain 76A (Viscardi et al., 2016; belonging to the microbial collection of the Department of Agricultural Sciences, University of Naples Federico II, Portici) and *Trichoderma afroharzianum* strain T22 (ex-*Trichoderma harzianum;* Cai and Druzhinina, 2021) isolated from the commercial formulation of Trianum-P (Koppert Biological Systems Rotterdam, the Netherlands) implemented at final concentration of 10^6^ spore mL^-1^. The inoculum preparation of the bacterial strain *A. chroococcum* 76A was performed according to Van Oosten et al. (2018).

Tomato seeds were surface-sterilized and coated with a microbial cell suspension containing *A. chroococcum* 76A (1 × 10^7^ CFU mL^-1^) and *T. afroharzianum* T22 (1 × 10^6^ spores mL^-1^) to uniformly cover the seed surface. Treated seeds were air-dried and hand-seeded in styrofoam planting trays containing a peat-based substrate for germination (Tecno Grow Semina 80, TerComposti SpA, Brescia, IT). At the time of transplant, one-month old tomato seedlings were inoculated with the microbial inoculum by using a root dip method, submerging the planting trays in the microbial liquid suspension for 15 min to completely wet the roots; drained of excess liquid, then the plant-plug was removed and transplanted to pre-bored holes in the soil at the field location. Further, at 15 and 45 DAT, each plant was repeatedly inoculated at the base with 50 mL of microbial suspension containing *A. chroococcum* 76A (1 × 10^7^ CFU mL^-1^) and *T. afroharzianum* T22 (1 × 10^6^ spores mL^-1^) (A+T). Uninoculated plants were treated only with water and served as control.

### 2.3 Plant growth and yield measurements

Plant growth parameters were evaluated at 45 Days After Transplant (DAT), at the flowering stage. Aboveground and belowground biomass was measured in terms of shoot fresh weight and root length and width. Five plants per treatment were cut at the soil surface, and the above-ground biomass was weighted on a balance for the evaluation of shoot fresh weight (FW). Root length and width were measured as previously described in Li et al. (2020), with minor modifications. Briefly, a soil trench, 70 cm deep and 60 cm wide, was excavated beside the plots to expose the soil profile of three plants per treatment, then maximum root length and width were measured. Yield parameters were evaluated at the end of the experiment (90 DAT; harvest). Tomato fruits were harvested for the determination of the fresh biomass and the number of fruits per plant. Brix degrees were measured with a bench refractometer (ATAGO palette - ATAGO CO., LTD – Japan).

### 2.4 Physiological parameters

Leaf water potential was measured at 45 DAT using a dewpoint psychrometer (WP4, Decagon Devices, Pullman, WA, USA) on fully expanded leaves. At the same date, leaf temperature was measured by thermometric measurements performed with a thermal IR camera (Seek CompactPRO, Seek Thermal, Inc. 6300 Hollister Ave - Santa Barbara, CA), and soil plant analysis development system (SPAD) with a portable SPAD-502 chlorophyll meter (Konica-Minolta, Tokyo, Japan).

### 2.5 Enumerations of microorganisms in the tomato rhizosphere

Viable microbial counts were performed at time of flowering (45 DAT) and at harvest (90 DAT), to assess the impact of the treatment with microbial consortia on the cultivable microbial community. Soil rhizosphere samples, 9 replicates for each treatment, were collected as previously reported (Romano et al., 2020b). Ten grams of rhizosphere composite samples (n=3) were suspended in 90 mL of quarter-strength Ringer’s solution (Oxoid, Milan, Italy). After shaking for 30 minutes, a dilution series was prepared in quarter strength Ringer’s solution, and aliquots were used to inoculate different solid and liquid media. Total heterotrophic aerobic bacteria were enumerated on Plate Count Agar (PCA; Oxoid, Milan, Italy) plates and incubated for 2 days at 28°C; whereas fungi were counted on Dichloran Rose Bengal Chloramphenicol Agar (DRBC, Oxoid) plates and incubated for 7 days at 28°C. To determine target microbial groups based on inoculum characteristics, free-living (N_2_)-fixing aerobic bacteria were counted on the Augier medium (Romano et al., 2020a), detecting a brown patina on surface of the liquid medium of positive tube after 15 days of incubation at 28°C; selective count of *Trichoderma* was performed as described by Caruso et al. (2020).

All tests were carried out in triplicate. Microbiological data were expressed as CFU or MPN g^-1^ of soil.

### 2.6 Molecular analysis of tomato rhizosphere

Total DNA was extracted from composite rhizosphere samples of tomato plants using a Fast DNA SPIN Kit for Soil (MP Biomedicals, Illkirch, France) according to the manufacturer’s instructions.

The primers V3f and V3r (Muyzer et al., 1993) were used to analyze prokaryotic populations. The primers NL1 (Kurtzman and Robnett, 1998) and LS2 (Cocolin, 2000) were employed for eukaryotic Denaturing Gradient Gel Electrophoresis (DGGE) analysis. A GC clamp was added to forward primers according to Muyzer et al. (1993). The PCR mixture and conditions for both amplifications were performed according to Di Mola et al. (2021). DGGE analyses were performed using a polyacrylamide gel [8% (wt/vol) acrylamide-bisacrylamide (37:5:1)] with a denaturing gradient of 30–60% by a Bio-Rad DCode Universal Mutation System (Bio-Rad Laboratories, Milan, Italy) as previously described (Ventorino et al., 2018). All tests were carried out in triplicate.

### 2.7 Statistical analysis

Agronomical and microbial counting data were analyzed by one-way ANOVA followed by Duncan’s *post hoc* test for pairwise comparison of means (at P < 0.05) using SPSS 21.0 statistical software package (SPSS Inc., Cary, NC, United States).

DGGE bands were automatically detect by Phoretix 1 advanced version 3.01 software (Phoretix International Limited, Newcastle upon Tyne, England). After the matching bands confrontation, a cluster analysis was performed as previously indicated by Ventorino et al. (2013). The correlation matrix of the band patterns was performed by using the method described by Saitou and Nei (1987). Finally, the percentage of similarity (S) of the microbial community was estimated by analyzing the resulting matrix using the average linkage method in the cluster procedure of Systat 5.2.1. According to Dong and Reddy, the structural diversity of the microbial community was examined by the Shannon index of general diversity H (Shannon and Weaver, 1963).

H was calculated on the basis of peak height from the different bacterial groups (16S rDNA bands) in the densitometric curve as indicated in the equation for the Shannon index:


$$\text{H}=-\;\sum \left({\text{n}}_{\text{i}}/\text{N})\log({\text{n}}_{\text{i}}/\text{N}\right)$$

where n_i_ is the height of the peak and N the sum of all peak heights of the densitometric curve. The analysis of band intensity was performed with GelAnalyzer 23.1.

## 3 Results

### 3.1 Plant growth and agronomic performance

**Figure 1:**
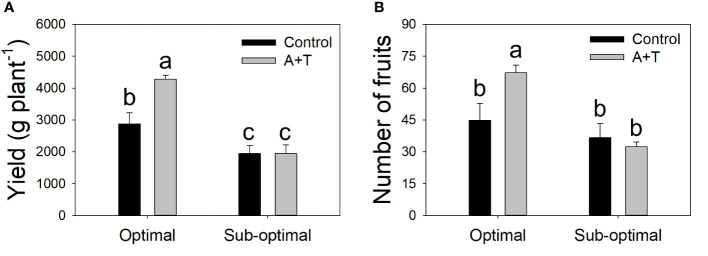
Yield **(A)** and number of fruits **(B)** of tomato plants grown under optimal and sub-optimal input (I), with or without (Control) the combined inoculum of *Azotobacter chroococcum* and *Trichoderma afroharzianum* (treatment A+T). Different letters indicate significant differences according to Duncan’s *post-hoc* test.

**Figure 2:**
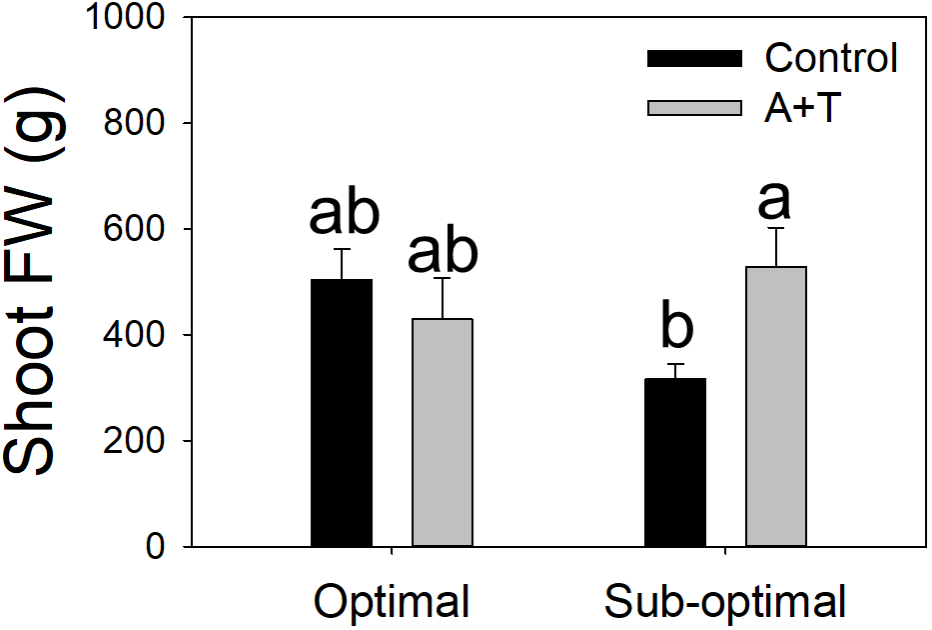
Shoot fresh weight of tomato plants grown under optimal and sub-optimal input (I), with or without (Control) the combined inoculum of *Azotobacter chroococcum* and *Trichoderma afroharzianum* (treatment A+T). Different letters indicate significant differences according to Duncan’s *post-hoc* test.

Evaluations of the overall yield indicated that both the Input (I; water and nitrogen fertilizer) and the Treatment (T; *Azotobacter* and *Trichoderma*) factors had a significant impact, with relevant differences (**Table 1**; **Figure 1**). Under optimal water and nutritional input, the microbial inoculum of *Azotobacter* and *Trichoderma* (A+T) increased by 48.6% and 50% tomato yield and number of fruits per plants, respectively (**Figure 1**). In contrast, conditions in water and nutrient shortage reduced the differences for both parameters. The decrease in the water and nutrient factors reduced plant above-ground and below-ground growth by about 10% (**Table 2**). Root treatments with the combined inoculum of *Azotobacter* and *Trichoderma* (A+T) enhanced the shoot fresh weight by about 15%, but resulted in a 16% and 20% decrease in the root length and width, respectively (**Table 2**). No effects of the input regime (water and nitrogen) were observed on this parameter. In contrast, a significant interaction “Input” x “Treatment” (IxT) was found in terms of aboveground plant biomass as indicated by shoot fresh weight (FW). Under optimal water/nitrogen conditions, the aboveground plant biomass of A+T treated plants was similar to untreated control plants (**Figure 2**). However, under sub-optimal water/nitrogen conditions, A+T plants had a 60% greater shoot biomass compared to untreated control plants (**Figure 2**).

**Table 1:** Productivity parameters of tomato plants grown under optimal and sub-optimal input (I) and with or without the combined inoculum of *Azotobacter chroococcum* 76A and *Trichoderma afroharzianum* T22 (treatment A+T).

	Yield	Number of fruits
	g plant^-1^	#
Input (I)		
Optimal	3580 a	56.0 a
Sub-optimal	1940 b	34.5 b
Treatment (T)		
Control	2410 b	40.7
A+T	3110 a	49.8
Interaction		
I	***	**
T	*	ns
IxT	*	*

Asterisks indicate significant differences according to ANOVA (ns, not significant; * = 0.05; ** = p<0.01; *** = p<0.001). Different letters after values indicate significant differences according to Duncan’s post-hoc test.

**Table 2:** Growth parameters of tomato plants grown under optimal and sub-optimal input (I), with or without (Control) the combined inoculum of *Azotobacter chroococcum* 76A and *Trichoderma afroharzianum* T22 (treatment A+T).

	Shoot FW	Root maximum length	Root maximum width
	g	cm	cm
Input (I)			
Optimal	466.5	36.65	32.00
Sub-optimal	423.2	35.25	33.15
Treatment (T)			
Control	410.5	39.90 a	37.75 a
A+T	479.2	33.55 b	30.05 b
Interaction			
I	ns	ns	ns
T	ns	***	**
IxT	*	ns	ns

Asterisks indicate significant differences according to ANOVA (ns, not significant; * = 0.05; ** = p<0.01; *** = p<0.001). Different letters after values indicate significant differences according to Duncan’s post-hoc test.

In terms of fruit quality, as determined by the measurement of Brix degree of sugar content, the low input regime increased by 28% the Brix score (3.46 under optimal vs. 4.08 under sub-optimal conditions), whereas no effect of the microbial treatment variable was detected (**Table S1**).

### 3.2 Physiological measurements

**Figure 3:**
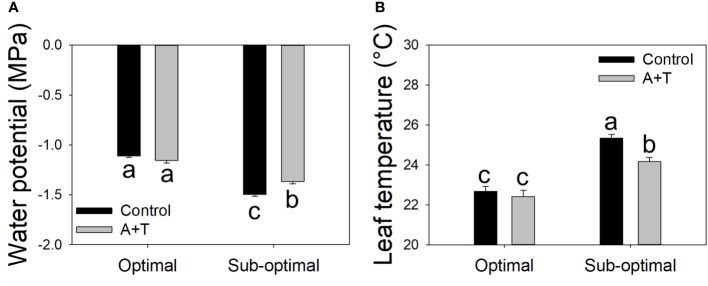
Leaf water potential **(A)** and leaf temperature **(B)** measurements of tomato plants grown under optimal and sub-optimal input (I), with or without the combined inoculum of *Azotobacter chroococcum* and *Trichoderma afroharzianum* (treatment A+T). Different letters indicate significant differences according to Duncan’s *post-hoc* test.

The results obtained from the physiological measurements of the plant water status were consistent with the trends observed in the evaluation of the plant growth parameters, whereby, the leaf water potential was similar in A+T treated *vs* control plants under optimal input (water and nitrogen). In contrast, under reduced input (water and nitrogen deficit) the leaf water potential was slightly, however significantly, higher in A+T inoculated plants compared to untreated plants (+9.5%; **Figure 3A**). These results were consistent with the leaf temperature of A+T inoculated plants such that under water and nitrogen deficit there was a significantly lower value measured compared to untreated plants (-4.6%; **Figure 3B**), and this corresponded to the aboveground biomass production, which was 40% higher in A+T inoculated plants compared to untreated control plants (**Figure 2**). In respect to the SPAD values, representative of the leaf chlorophyll content, the low input treatment (I) decreased the value by 10.7% compared to optimal cultural conditions, whereas the microbial treatment increased the SPAD value by 4.9% compared to untreated control. No interaction between I and T factors was found (**Table 3**).

**Table 3:** Physiological parameters of tomato plants grown under optimal and sub-optimal input (I) and with or without the combined inoculum of *Azotobacter chroococcum* 76A and *Trichoderma afroharzianum* T22 (treatment A+T).

	Leaf water potential	SPAD	Leaf temperature
	MPa		°C
Input (I)			
Optimal	-1.14 a	57.8 a	22.5 b
Sub-optimal	-1.47 b	51.6 b	24.7 a
Treatment (T)			
Control	-1.32	53.4 b	24.0 a
A+T	-1.29	56.0 a	23.3 b
Interaction			
I	***	***	***
T	ns	*	**
IxT	***	ns	*

Asterisks indicate significant differences according to ANOVA (ns, not significant; * = 0.05; ** = p<0.01; *** = p<0.001). Different letters after values indicate significant differences according to Duncan’s post-hoc test.

### 3.3 Enumerations of microorganisms in the tomato rhizosphere

Significant differences in the total heterotrophic aerobic bacteria were found between inoculated and non-inoculated plots at flowering and harvesting phase (**Table 4**). In optimal conditions, the microbial concentration was lower in inoculated plots compared to non-inoculated plots at time of flowering, However, in sub-optimal conditions, a significant increase (ca. 1 Log) in these populations was noted. In this case, the microbial population in the rhizosphere of inoculated plants (7.73±0.04 Log CFU g^-1^) was greater than the control (6.70±0.22 Log CFU g^-1^). Suggesting that the applied microbial inoculum may exert a positive effect on soil microflora especially under stress conditions. By contrast, at the end of experiment, total heterotrophic aerobic bacteria in the treated plots were significantly higher than in the non-inoculated control, whereas no significant differences were found in sub-optimal plots (**Table 4**).

**Table 4:** Enumerations (log CFU or MPN g^− 1^) of total heterotrophic aerobic bacteria, molds, free-living (N_2_)-fixing aerobic bacteria and *Trichoderma* in the rhizosphere of tomato plants grown under optimal and sub-optimal input, without microbes (Control) or with the *Azotobacter chroococcum* 76A and *Trichoderma afroharzianum* T22 inoculum (treatment A+T) collected at phenological stages at time of flowering or harvest.

	Treatment	Sampling	Input	
			Optimal	Sub-optimal
**Total heterotrophic aerobic** **bacteria**	Control	Flowering	7.72 ± 0.02 a	6.70 ± 0.22 de
		Harvest	6.38 ± 0.04 g	6.88 ± 0.02 cd
	A+T	Flowering	7.07 ± 0.04 bc	7.73 ± 0.04 a
		Harvest	6.59 ± 0.01 e	6.87 ± 0.34 cd
**Moulds**	Control	Flowering	4.82 ± 0.01 c	4.79 ± 0.01c
		Harvest	3.74 ± 0.04 e	4.58 ± 0.08 d
	A+T	Flowering	4.93 ± 0.01 b	6.40 ± 0.08 a
		Harvest	4.73 ± 0.00 c	4.58 ± 0.08 d
**N_2_-fixers**	Control	Flowering	2.65 ± 0.00 c	2.98 ± 0.00 bc
		Harvest	1.98 ± 0.00 e	1.98 ± 0.00 e
	A+T	Flowering	3.15 ± 0.12 b	3.82 ± 0.16 a
		Harvest	2.32 ± 0.09 d	2.32 ± 0.14 d
***Trichoderma***	Control	Flowering	2.46 ± 0.15 cd	2.59 ± 0.11 c
		Harvest	2.25 ± 0.24 de	2.20 ± 0.17 e
	A+T	Flowering	3.57 ± 0.01 b	3.74 ± 0.02 b
		Harvest	3.71 ± 0.01 b	4.01 ± 0.01 a

The measurement of the various microorganisms was determined by growth on diverse selective solid substrates indicated in the methods. Different letters after values indicate significant differences (p < 0.05) according to Duncan’s post-hoc test.

Similarly, the microbial inoculum also affected the fungal community. In fact, a significant increase in fungal counts was detected in the rhizosphere of inoculated plants (in the range of 4.58±0.08 - 6.40±0.08 Log CFU g^-1^), in respect to the non-inoculated control (in the range of 3.74±0.04 - 4.82±0.01 Log CFU g^-1^) in all conditions except for the condition in the sub-optimal environment at harvesting stage (**Table 4**).

Finally, at flowering, a significant increase, almost 1 Log CFU g^-1^, in the free-living (N_2_)-fixing bacteria was revealed in the rhizosphere of treated plants compared to non-inoculated plants subject to stress conditions (**Table 4**). At the end of experiment, although a drastic reduction was observed in all samples, N_2_-fixers were always significantly higher in the inoculated plants cultivated under sub-optimal conditions in respect to the non-inoculated samples (**Table 4**). The CFU of *Trichoderma* showed a positive trend (> 1 Log CFU g^-1^) in the rhizosphere of plants treated with microbial consortium compared to indigenous *Trichoderma* spp. of untreated plants. Moreover, at harvest a consistent decrease in indigenous *Trichoderma* spp. count was recorded from untreated plants, while a significant increase of *Trichoderma* abundance was observed in treated plants in sub-optimal conditions (**Table 4**).

### 3.4 Molecular characterization of soil microbes in tomato rhizosphere under optimal or stress conditions

**Figure 4:**
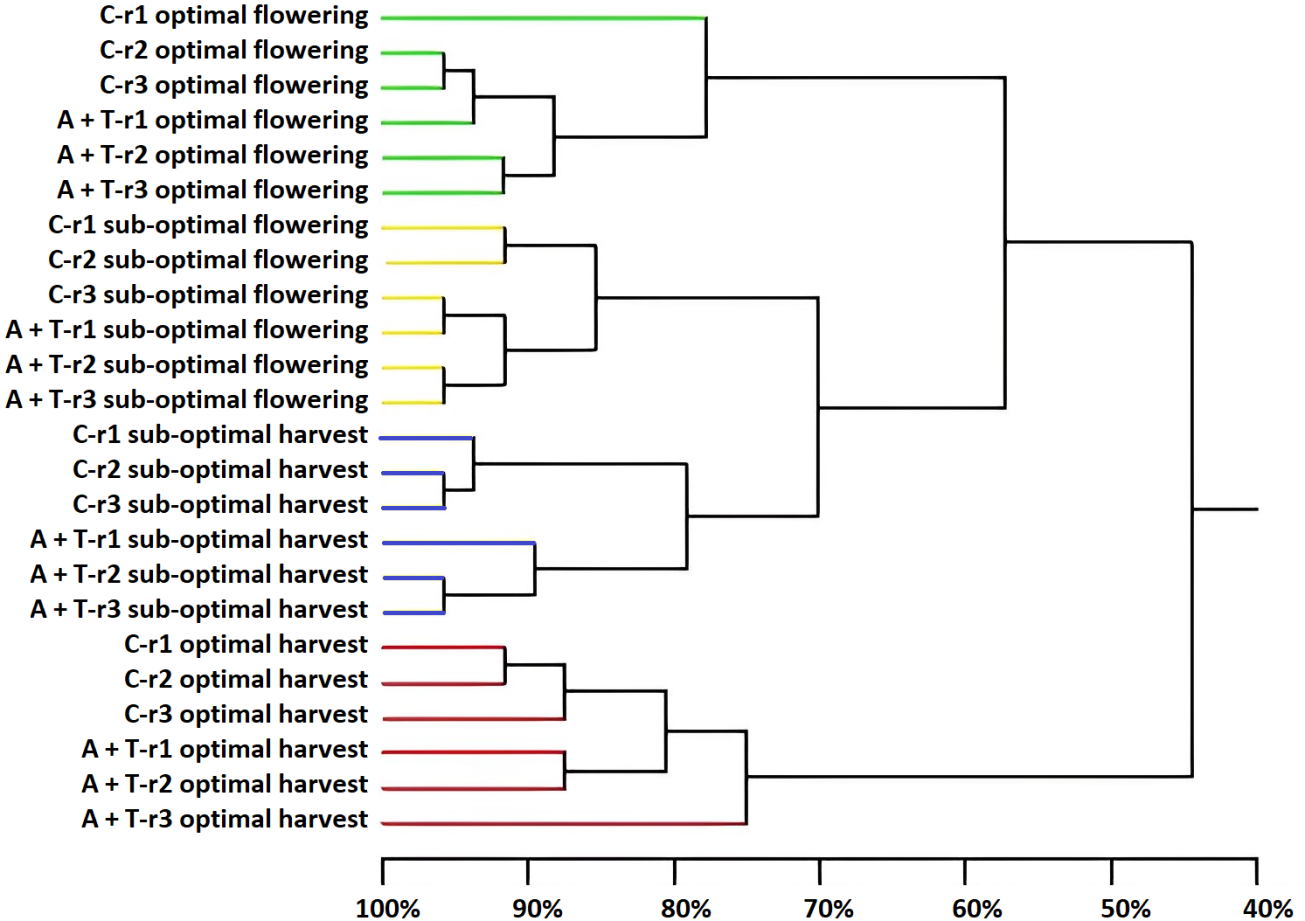
Dendrogram showing the degree of similarity (%) of the PCR-DGGE profiles of bacterial populations in the rhizosphere of tomato plants grown under optimal and sub-optimal input (I), without (C) or with the combined inoculum of *Azotobacter chroococcum* 76A and *Trichoderma afroharzianum* T22 (treatment A+T) collected at flowering or harvest stage, from three different replicates r1, r2 and r3. Different colors indicate different major clusters.

**Figure 5:**
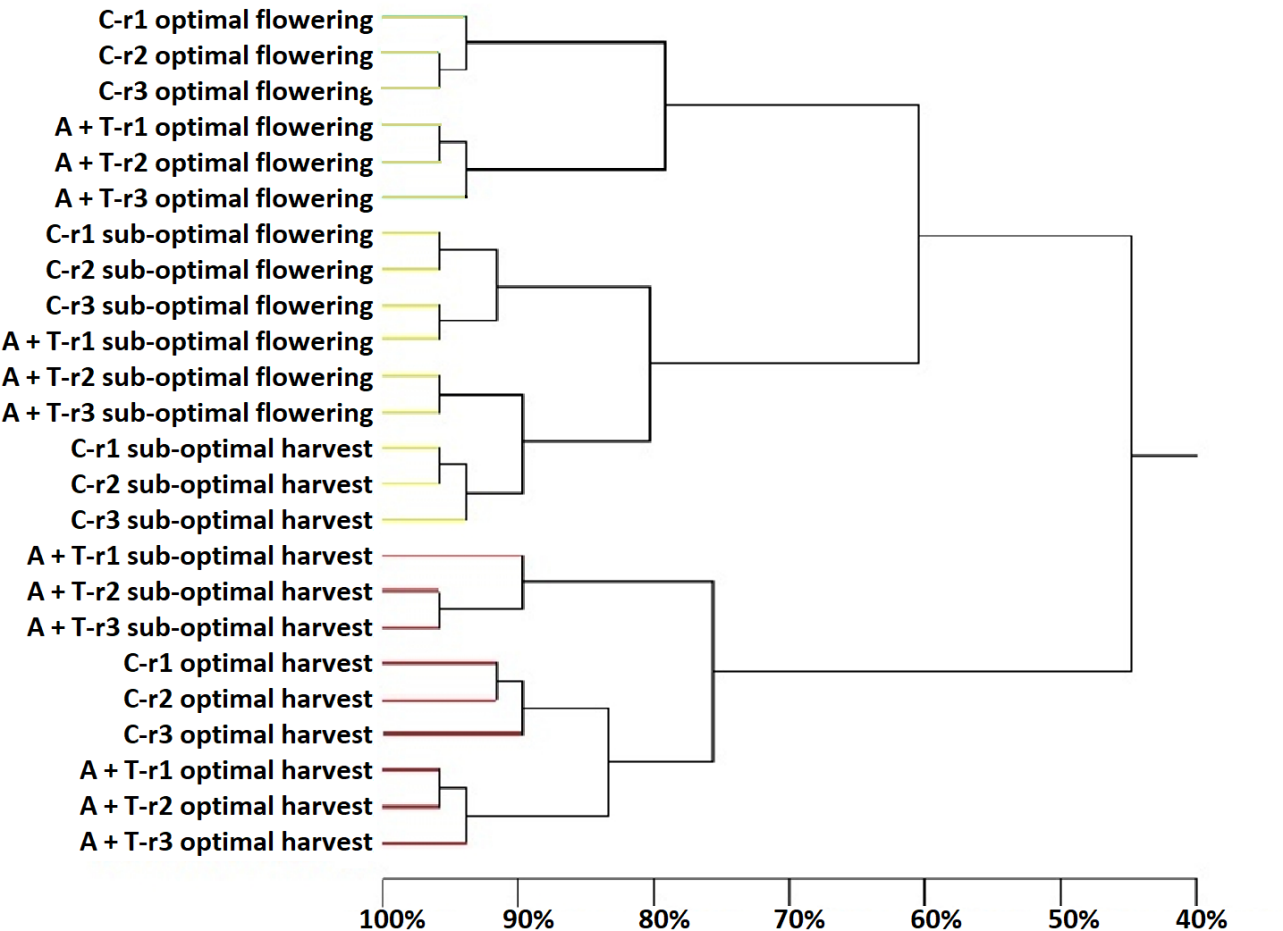
Dendrogram showing the degree of similarity (%) of the PCR-DGGE profiles of fungal populations in the tomato rhizosphere samples in the rhizosphere of tomato plants grown under optimal and sub-optimal input (I), without (C) or with the combined inoculum of *Azotobacter chroococcum* 76A and *Trichoderma afroharzianum* T22 (treatment A+T) collected at flowering or harvest stage, from three different replicates r1, r2 and r3. Different colors indicate different major clusters.

PCR-DGGE was employed to obtain a qualitative fingerprint of the bacterial and fungal communities in the tomato rhizosphere receiving to the combined application effects of abiotic stress (water and nitrogen) and microbial inoculation. The main results indicated that the sampling time was the major determinant of the composition and structure of the bacteria and fungi because it, more than the cultivation conditions and inoculum, determined the clustering into groups (**Figures 4**, **5**).

The DGGE profiles of the bacterial populations in the tomato rhizosphere were complex, producing 20-22 and 17-20 bands in inoculated samples and non-inoculated controls, respectively. Patterns indicated that microbial inoculum affected the richness of bacterial populations since the number of DGGE bands was significantly higher in the inoculated (A+T) than non-inoculated (C) plants (**Table S2**). Furthermore, the interaction between the sampling time and inoculum played a key role in affecting the bacterial biodiversity demonstrating that the rhizosphere of tomato plants co-inoculated with *A. chroococcum* 76A and *T. afroharzianum* T22 showed a number of bands higher than non-inoculated plants (**Table S2**).

Differences in the samples due to the position and intensity of the bands were evaluated by statistical analysis. It was apparent that sampling time was the main driver in determining the prokaryotic diversity. Cluster analysis (**Figure 4**) identified four major groups associated to the sampling time and cultivation conditions (cluster 1: samples cultivated in optimal conditions and collected at flowering; cluster 2: samples cultivated in sub-optimal conditions and collected at flowering; cluster 3: samples cultivated in sub-optimal conditions and collected at harvesting; cluster 4: samples cultivated in optimal conditions and collected at harvesting). Clusters 2 and 3, comprising the rhizosphere samples obtained from tomato plants cultivated in stress conditions, were very similar, demonstrating a similarity of 70%; while cluster 1 had a similarity as low as 57% with the assembly of these two groups and cluster 4 was only 45% similar to these groups (**Figure 4**). However, within each of the major clusters, the sub-groupings of the bacterial populations were always similar and determined by the microbial inoculum applications with a high similarity level that ranged from 76% to 85% (**Figure 4**). The Shannon-Weaver index of bacterial populations was significantly affected by input (I), microbial treatment (T), and sampling time (ST), as well as by the interaction of the input for the phenological stage (IxST; **Table 5**). Specifically, this index was higher in sub-optimal conditions, in the presence of the A+T inoculum, and during the flowering stage. These findings were also observed in the interaction between input and phenological stage, with a higher value in sub-optimal conditions during the flowering stage (**Table 5**).

**Table 5:** Means and standard deviations of the Shannon diversity index (H) based on DGGE bands intensity of bacterial and fungal populations in the rhizosphere of tomato plants grown under optimal and sub-optimal inputs (I), without microbes (C) or treated with the microbial inoculum (T) of *Azotobacter chroococcum* 76A and *Trichoderma afroharzianum* T22 (A+T) collected at sampling times (ST) of flowering or at harvest.

	Source of Variance	Shannon Index Bacteria	Shannon Index Fungi
Input (I)	Optimal	0.91 ± 0.08^b^	0.78 ± 0.19
	Sub-optimal	1.05 ± 0.18^a^	0.61 ± 0.29
		***	ns
Treatment (T)	C	0.94 ± 0.17^b^	0.71 ± 0.28
	A+T	1.02 ± 0.13^a^	0.69 ± 0.24
		*	ns
Sampling Time (ST)	Flowering	1.05 ± 0.17^a^	0.73 ± 0.27
	Harvest	0.90 ± 0.09^b^	0.67 ± 0.25
		***	ns
IxC	Optimal x C	0.87 ± 0.09	0.86 ± 0.13
	Optimal x A+T	0.94 ± 0.04	0.70 ± 0.22
	Sub-optimal x C	1.00 ± 0.21	0.53 ± 0.32
	Sub-optimal x A+T	1.09 ± 0.15	0.68 ± 0.27
		ns	
TxST	C x flowering	1.03 ± 0.19	0.63 ± 0.31^ab^
	C x harvest	0.85 ± 0.09	0.81 ± 0.22^a^
	A+T x flowering	1.07 ± 0.17	0.84 ± 0.17^a^
	A+T x harvest	0.96 ± 0.03	0.55 ± 0.21^b^
		ns	*
IxST	Optimal x flowering	0.89 ± 0.08^b^	0.77 ± 0.16
	Optimal x harvest	0.92 ± 0.08^b^	0.79 ± 0.23
	Sub-optimal x flowering	1.20 ± 0.04^a^	0.69 ± 0.35
	Sub-optimal x harvest	0.89 ± 0.10^b^	0.52 ± 0.19
		***	ns
IxTxST	Optimal x C x flowering	0.87 ± 0.11	0.77 ± 0.10
	Optimal x C x harvest	0.97 ± 0.05	0.94 ± 0.12
	Optimal x A+T x flowering	0.92 ± 0.03	0.77 ± 0.24
	Optimal x A+T x harvest	0.97 ± 0.05	0.63 ± 0.23
	Sub-optimal x C x flowering	1.18 ± 0.03	0.48 ± 0.41
	Sub-optimal x C x harvest	0.82 ± 0.10	0.62 ± 0.21
	Sub-optimal x A+T x flowering	1.22 ± 0.03	0.90 ± 0.08
	Sub-optimal x A+T x harvest	0.96 ± 0.02	0.46 ± 0.18
		ns	ns

24/11/2023 4:20:45 pmAsterisks indicate significant differences according to ANOVA (ns, not significant; * = 0.05; ** = p<0.01; *** = p<0.001). Different letters within each column indicate significant differences according to Duncan’s post-hoc test.

DGGE of the fungal populations showed a low complex profile producing a number of bands ranging from 11 to 15. However, fungal diversity was affected by several parameters. The number of bands of fungal populations was significantly affected by microbial inoculum applications since the number of DGGE bands was higher in the inoculated than non-inoculated plants (**Table S2**). The number of fungal bands was significantly higher under optimal than sub-optimal growth conditions. Finally, the sampling time also affected fungal biodiversity showing higher values at harvesting than flowering (**Table S2**).

As shown in the **Figure 5**, statistical analysis on the position and intensity of the bands allowed the classification of two major clusters clearly associated to the two sampling times with a similarity level of 46% (cluster 1: all the samples collected at flowering stage and non-inoculated plants cultivated in sub-optimal conditions and collected at harvesting; cluster 2: samples inoculated with microbial strains and collected at harvesting and non-inoculated control samples cultivated in optimal conditions and collected at harvesting). It was interesting to note that within each of the major clusters delineated by the sampling time, the sub-groupings of the eukaryotes were similar and firstly determined by the stress conditions and then by microbial inoculum applications. In fact, three sub-clusters, with a high similarity level ranging from 76% to 81%, were delineated by cultivation conditions (optimal or suboptimal). Moreover, within these groups’ other sub-groupings of the fungi were always determined by the microbial inoculum (similarity level ranging from 90% to 95%; **Figure 5**). Nevertheless, the Shannon-Weaver index of fungal populations was influenced by the interaction between microbial inoculum and sampling time (**Table 5**); indeed, Shannon index values were higher with microbial inoculum at flowering.

## 4 Discussion

### 4.1 Simultaneous application of *Trichoderma* and *Azotobacter* enhances yield in tomato and alleviates combined water-nitrogen stress

Plant biostimulants, including microorganisms such as fungi and PGPR, have been increasingly used to help crops to tolerate and/or adapt to environmental stress (Li et al., 2022; Gul et al., 2023). Microorganisms of diverse origin have been proven to protect plants from water deficit (Silletti et al., 2021), temperature extremes (Shaffique et al., 2022), salinity (Van Oosten et al., 2018), pathogens (Khan et al., 2020) and other biotic factors (Woo et al., 2023). However, most published literature refers to plant protection upon exposure to single stress, whereas how microorganisms and/or biostimulants in general could facilitate plant adaptation to multiple abiotic stresses has rarely been addressed. Coexistence of multiple stresses is a much more frequent occurrence both in nature and agricultural systems (Kissoudis et al., 2014) and it may require the need of more complex formulations of biostimulants and beneficial microorganisms, able to simultaneously potentiate different physiological responses of the plant to specific stresses (Parađiković et al., 2019) or activate multiple resistance mechanisms (Woo et al., 2023). In our previous work (Silletti et al., 2021), it was demonstrated that although biostimulants may be capable of enhancing growth and stress tolerance, the soil nutrient availability and environmental conditions may heavily influence these responses. Furthermore, it was also shown that *T. afroharzianum* strain T22 acted mostly as a growth enhancer under optimal irrigation and moderate drought stress (50% replenishment of plant water requirements), whereas *A. chroococcum* strain 76A improved plant water relations under stronger stress conditions (25% replenishment of plant water requirements). Based on these results, it was hypothesized that together *T. afroharzianum* strain T22 and *A. chroococcum* strain 76A could reinforce the protective action not only to single abiotic factors, but also to diverse combinations of multiple stresses (water shortage and sub-optimal N availability) since these two strains were likely acting via different plant-microbe interaction mechanisms (Woo and Pepe, 2018). Such microbial consortium could offer a strategy to respond to the urgent challenges posed by sustainable agriculture and global food demand (van Dijk et al., 2021). A reduced water and nitrogen availability resulted in a 50% yield reduction in the untreated control plants, confirming that these plants were operating in sub-optimal water-nitrogen regime. The A+T treatment increased yield by 48.6% under optimal conditions, however the mixture was not able to compensate the effects of water and nitrogen shortage (**Figure 1A**). Treatments with *Trichoderma* spp. and *A. chroococcum* have been proven to have variable effects from other general growth enhancers (Di Mola et al., 2023), and to be more protectant to specific stress (Woo et al., 2023). This may also be a consequence of multiple, variable and complex interactions that plants establish with the surrounding environment, and not only the microbial component (Del Buono, 2021; Silletti et al., 2021). The activation of various biosynthesis functions in the plant have been attributed to *Trichoderma* interactions with the host, such as the activation of the antioxidant machinery (Mastouri et al., 2012), the regulation of phytohormones (Illescas et al., 2021), and the solubilization of phosphate and micronutrients (Li et al., 2015). Plant growth promotion effects by *Trichoderma* spp. have been noted in the increased root biomass in some crops (Macías-Rodríguez et al., 2018; Sehim et al., 2023). However, our results indicated that in combination with A+T there was a reduction in the root length and width (**Table 2**).

Similarly, Prajapati et al. (2008) have noted that a bacterial treatment with *A. chroococcum* in rice caused a significant decrease in root dry weight as compared to control plants. This limitation in root architecture may serve to assist the plant in tolerating the environmental stress conditions in the soil, that results in the redistribution of necessary resources to other vegetative structures. Most interestingly in our present work, the reduced root expansion due to the A+T treatment did not affect yield under sub-optimal conditions, and it was actually positively correlated to an improved yield under optimal conditions (**Figure 1**). Similarly, the higher SPAD values in A+T treated plants compared to control plants (**Table 3**) may indicate an improved nutritional status A+T plants, since SPAD values are correlated with the nitrogen status of the plant (Ghosh et al., 2023). This suggests that the microorganisms may increase nutrient availability in the rhizosphere, for example acting as siderophores or biodegraders, working in the conversion of iron, zinc or phosphorus elements into forms utilizable by the plant (Woo and Pepe, 2018; Woo et al., 2023). A+T treatments are known to improve plant tolerance to abiotic stress (Silletti et al., 2021), possibly by increasing plant root efficiency in terms of water and nitrogen uptake and/or enhancing the absorption and assimilation of water and nitrogen in the root zone. Although the physiological basis of these effects is unclear, it is at least consistent with the higher carbon allocation to root expansion in response to nutrient and water shortage (Bicharanloo et al., 2023; Wang L. et al., 2023), which is not sensed in A+T treated plants (**Table 2**). Moreover, an improved leaf water potential of treated plants under sub-optimal growth conditions was observed (**Figure 3A**) that corresponded to lower leaf temperatures (**Figure 3B**), and higher shoot fresh weight (**Figure 2**). This may indicate that the microbial consortium can act as positive regulator of plant water relations, perhaps by cooling the temperatures in the leaf reduces the physiological processes that limit transpiration and the rate of water loss by the plant particularly under limited water and nitrogen availability. Although this response was not sufficient to ameliorate plant yield under sub-optimal conditions, the positive A+T effect was clear under optimal conditions in terms of yield and fruit number (**Figure 1**). This was likely associated to a reallocation of plant biomass from roots to reproductive organs (Eziz et al., 2017) that may have been triggered by the A+T treatment. The higher SPAD of A+T treated plants under both input levels also confirmed an improved nutritional status of these plants. Overall, the effects of the microbial treatment appeared to have altered the physiological mechanisms that mediate tomato yield and stress adaptation in a fashion that deserves further investigation.

### 4.2 Rhizosphere microbial diversity is improved by *Trichoderma afroharzianum* T22 and *Azotobacter chroococcum* 76A co-inoculation in agricultural soils

Due to the close interactions with the surroundings and the high surface area to volume ratio, soil microbiota could be particularly sensitive to environmental stresses and soil perturbations compared to higher organisms (Karimi et al., 2017; Gugliucci et al., 2023). By using cultural methods, it was possible to monitor the significant impact of the inoculation with the *T. afroharzianum* T22 and *A. chroococcum* 76A consortium on the indigenous soil microbiota in the rhizosphere of tomato plants, that included heterotrophic aerobic bacteria populations, free-living (N_2_)-fixing bacteria, fungi including *Trichoderma* spp. A notable increase in the microbial populations was observed in the combined stress conditions, indicating the potential of microbial inoculants to enhance the native soil microbiota abundance, possibly the beneficial microorganisms. In line with this observation, it was noted that the Shannon diversity index exhibited higher values in the rhizosphere inoculated with the microbial consortium compared to the control, especially within bacterial populations. Whereas, for the fungal community, the effect depended on the interaction between microbial inoculum and sampling time. Similar to findings in previous research, this work has demonstrated that soil inoculation with selected microorganisms or microbial consortia can induce significant alterations in both bacterial and fungal communities (Fiorentino et al., 2018; Ventorino et al., 2018; Chouyia et al., 2020). Furthermore, the application of a *T. afroharzianum* T22 and *A. chroococcum* 76A consortium to wheat plants cultivated under stress conditions has shown a remarkable capacity to positively influence and improve the microbial community effects on the agronomic characteristics of the crop (Silletti et al., 2021). This evidence indicates a great application potential for using a microbial consortium on various crops in order to enhance microbial concentrations within the rhizosphere that also includes augmenting the presence and activities of the native beneficial microorganisms. In fact, inoculated microorganisms may synergistically collaborate within the rhizosphere, forming complex networks of interactions that affect microbial community composition and structure, resulting in beneficial outcomes for plant growth and development (Santoyo et al., 2021). Unlike single microbial inoculants, microbial consortia offer additional benefits through their wide range of functions (Ju et al., 2019) which could enhance the strength and productivity of the whole microbiota (Santoyo et al., 2021). Thus, the application of microbial consortia plays a key role in shaping and enhancing microbial communities within agricultural ecosystems, which in turn, have a significant impact on the fertility of agricultural soils and influence ecosystem function and productivity (Ventorino et al., 2019b). By harnessing the collective capabilities of multidisciplinary interacting microorganisms, these consortia promote sustainable agriculture by bolstering plant growth, reducing the dependency on agrochemicals, and preserving the health and equilibrium of the soil microbiota (Woo and Pepe, 2018; Santoyo et al., 2021; Woo et al., 2023).

Our results also highlighted the impact of sampling time as an important factor determining the composition and structure of either bacterial or fungal communities in the tomato rhizosphere. Several studies have demonstrated that the phenological stages of plant development have a great influence on microbial communities in plant-soil compartment niches (Xiong et al., 2021; Ajilogba et al., 2022). DGGE analysis revealed that both bacterial and fungal populations in the tomato plant rhizosphere exhibited differences primarily attributed to the diverse sampling times at flowering or harvest, followed by the effects of the water and nitrogen inputs, and finally the influence by the microbial inoculum application. This suggests that the impact of microbial consortium is modulated by the existing stressors in the cultivation environment, highlighting the need to understand the relationship between stress factors and microbial communities in agricultural soils. The shifts of climatic factors, such as temperature and precipitation, during seasons are often the strongest factors influencing microbial composition and dynamics (Cruz-Martínez et al., 2009; Xue et al., 2011). In open field trials, both biotic and abiotic factors, such as the presence of microbial antagonists (e.g., protists or nematodes) and the availability of a carbon source, could influence the soil microbial community composition (Fierer, 2017). On the other hand, a temporal shift in rhizosphere microbial community may be attributed to the plant interactions with specific microorganisms at a given moment, and these interactions will vary as the plant grows, be influenced by compounds released by the host such as root exudates that shape the surrounding microbiome (Santoyo et al., 2021).

However, in the tomato rhizosphere, microbial diversity was also related to nitrogen and water inputs. Nitrogen treatments have been shown to exert distinct plant-mediated effects, leading to changes in the microbial communities living in the rhizosphere (Ramirez et al., 2010; Liu et al., 2021). Different nitrogen levels have proven to have a significant impact on the distribution and composition of bacterial communities in plant monocultures such as lettuce and rocket (Li et al., 2016; Fiorentino et al., 2018). Furthermore, N fertilization may directly or indirectly alter the soil microbiome by decreasing bacterial diversity and shifting toward a more active and copiotrophic microbial community (Li et al., 2021). Wang X. et al. (2023) revealed significant alterations in the soil microbiota structure due to N fertilization likely due to the microbial adaptation to N-excess although without significant effect on microbial richness and beta-diversity. Furthermore, application of N fertilizers can stimulate the production of plant root exudates that can enhance nutrient utilization by microbiota, as previously suggested by Sørensen (1997). However, the response of agroecosystem microbiota to N fertilization can change, leading to unpredictable outcomes for nitrogen-fixing activity in the rhizosphere, as emphasized by Saraf et al. (2011).

Water input can also modify both the composition and activity of soil microbial communities, since changes in soil water content could affect the availability of soil nutrients (Li et al., 2021). Yuan et al. (2016) observed that irrigation practices had a stronger effect on the abundance, diversity, and structure of bacterial communities than fertilization, confirming the driving effect of soil moisture on shift of bacterial communities. Recently, Xu et al. (2020) observed that microbial community composition was affected by changes in water availability, showing that drought generally led to a decline in microbial biomass, while enhanced irrigation resulted in an increase, which might further translate into changes in microbial community composition (Romano et al., 2023).

## 5 Conclusions

Although the use of microbial-based biostimulants to aid crops in overcoming and/or adapting to single environmental stresses have been widely studied in recent years, little is known about how microbial consortia could facilitate plant tolerance to multiple stresses, a situation that is much more frequently encountered in both natural and agricultural systems. The interactions among microorganisms and between microorganisms and plants in the soil environment are complex due to various factors that determine the colonization and proliferation of these components, including overlapping needs and competition effects, the variability of field conditions, and/or other environmental stressors that may affect a functional agroecosystem equilibrium. The development of efficient and stable multipurpose microbial consortia requires holistic investigations that address such complexity under variable field conditions, including the co-existence of multiple stresses to which crops are generally exposed. This work advances our knowledge on a new *Azotobacter* and *Trichoderma*-based inoculum, its effects on the native microbial communities and on tomato plant responses to combined water and nitrogen deficiency. The overall results demonstrate this specific consortium had significant growth and yield enhancing properties on tomato and suggest that, in low-input cropping systems, it may help to cope with environmental constraints and limited chemical fertilization.

## Data availability statement

The original contributions presented in the study are included in the article/**Supplementary Material**, further inquiries can be directed to the corresponding author/s.

## Author contributions

VC: Formal analysis, Writing – original draft. IR: Formal analysis, Investigation, Writing – original draft. SW: Writing – review & editing, Investigation. ED: Investigation, Writing – review & editing. NL: Formal analysis, Writing – review & editing. EC: Formal analysis, Writing – review & editing. OP: Writing – review & editing, Investigation. VV: Conceptualization, Supervision, Writing – review & editing, Investigation. AM: Writing – review & editing, Investigation.
