# Supplementary material for: Inoculation with a microbial consortium increases soil microbial diversity and improves agronomic traits of tomato under water and nitrogen deficiency
Source: Front Plant Sci. 2023 Dec 6;14:1304627. doi: 10.3389/fpls.2023.1304627 (PMC10731302; doi:10.3389/fpls.2023.1304627)
Supplement: Supplementary file 1 [file DataSheet_1.pdf]

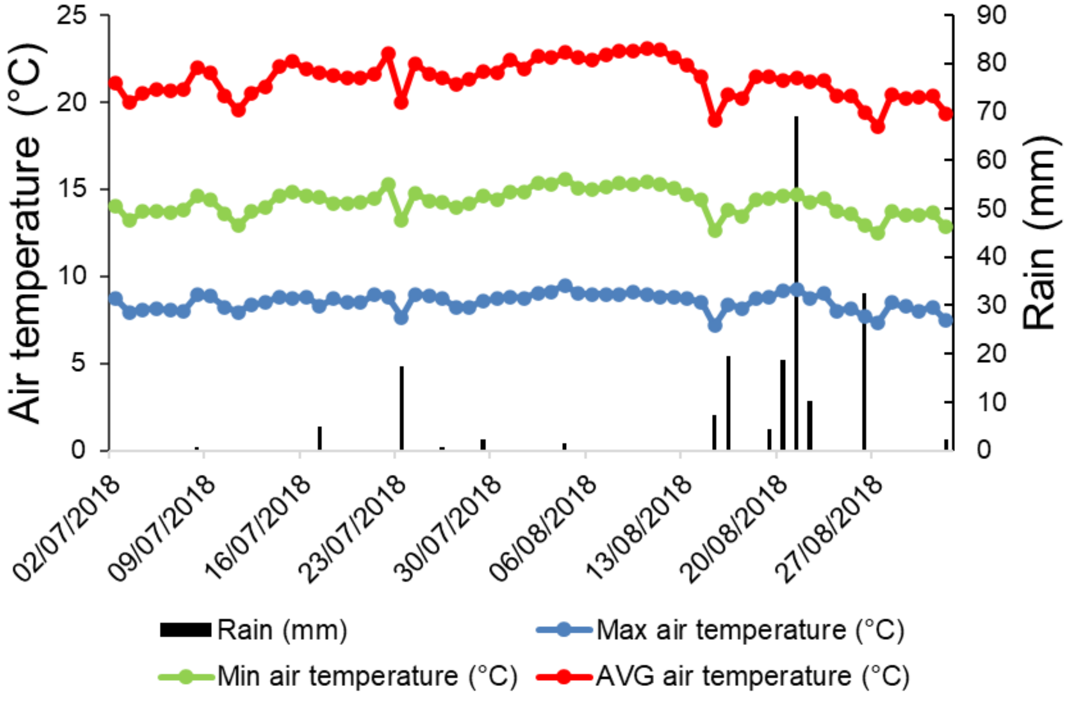

Figure S1. Meteorological data (min, max, avg temperature and rain) during the cultivation period of tomato in Bellizzi.

Table S1. Brix degrees of tomato plants grown under optimal and sub-optimal input (I) and with or without the combined inoculum of *Azotobacter chroococcum* 76A and *Trichoderma afroharzianum* T22 (treatment A+T). Asterisks indicate significant differences according to ANOVA (ns = not significant; ** = p<0.01).

	**Brix** degrees
Input (I)	
Optimal	3.46 b
Sub-optimal	4.08 a
Treatment (T)	
Control	3.94
A+T	3.60
Interaction	
I	**
T	ns
IxT	ns

Table S2. Statistical analysis of DGGE bands of bacterial and fungal populations in the rhizosphere of tomato plants grown under optimal and sub-optimal inputs (I), without microbes (C) or treated with the microbial inoculum (T) of *Azotobacter chroococcum* 76A and *Trichoderma afroharzianum* T22 (A+T) collected at sampling times (ST) of flowering or at harvest. Asterisks indicate significant differences according to ANOVA (ns = not significant; * = 0.05; ** = p<0.01; *** = p<0.001). Different letters within each column indicate significant differences according to Duncan's post-hoc test.

	**Source of Variance**	**n. of bands Bacteria**	**n. of bands Fungi**
Input (I)	Optimal	19.33 ± 0.43	14.25 ± 0.25 a
	Sub-optimal	19.83 ± 0.30	13.83 ± 0.24 b
		Ns	***
Treatment (T)	C	18.58 ± 0.26 b	13.50 ± 0.15 b
	A+T	20.58 ± 0.19 a	14.58 ± 0.23 a
		***	***
Sampling Time (ST)	Flowering	19.33 ± 0.45	13.58 ± 0.26 b
	Harvest	19.83 ± 0.27	14.50 ± 0.15 a
		ns	***
IxC	Optimal x C	18.17 ± 0.40	13.50 ± 0.22 b
	Optimal x A+T	20.50 ± 0.34	15.00 ± 0.00 a
	Sub-optimal x C	19.00 ± 0.26	13.50 ± 0.22 b
	Sub-optimal x A+T	20.67 ± 0.21	14.17 ± 0.40 b
		ns	***
TxST	C x flowering	18.00 ± 0.37 c	13.00 ± 0.00
	C x harvest	19.17 ± 0.17 b	14.00 ± 0.00
	A+T x flowering	20.67 ± 0.21 a	14.17 ± 0.40
	A+T x harvest	20.50 ± 0.34 a	15.00 ± 0.00
		*	Ns
IxST	Optimal x flowering	18.83 ± 0.70	14.00 ± 0.45 ab
	Optimal x harvest	19.83 ± 0.48	14.50 ± 0.22 a
	Sub-optimal x flowering	19.83 ± 0.54	13.17 ± 0.17 b
	Sub-optimal x harvest	19.83 ± 0.31	14.50 ± 0.22 a
		ns	***
IxTxST	Optimal x C x flowering	17.33 ± 0.33	13.00 ± 0.00 c
	Optimal x C x harvest	19.00 ± 0.00	14.00 ± 0.00 b
	Optimal x A+T x flowering	20.33 ± 0.33	15.00 ± 0.00 a
	Optimal x A+T x harvest	20.67 ± 0.67	15.00 ± 0.00 a
	Sub-optimal x C x flowering	18.67 ± 0.33	13.00 ± 0.00 c
	Sub-optimal x C x harvest	19.33 ± 0.33	14.00 ± 0.00 b
	Sub-optimal x A+T x flowering	21.00 ± 0.00	13.33 ± 0.33 c
	Sub-optimal x A+T x harvest	20.33 ± 0.33	15.00 ± 0.00 a
		ns	***
